# Call for a Global Vaccine Plan to Combat Current and Future Pandemics: One for ALL and ALL for One

**DOI:** 10.2174/18743064-v16-e2202040

**Published:** 2022-03-15

**Authors:** Salim Surani, Pahnwat T. Taweesedt, Sara Surani, Iqbal Ratnani, Joseph Varon

**Affiliations:** 1 Medicine & Pharmacology Texas A&M University, College Station, Texas, 77843, USA Research Collaborator, Mayo Clinic, Rochester, MN USA; 2 Pulmonary Associates Corpus Christi, Texas, 78404, USA; 3 Tsinghua University, Global Affairs, Beijing, China Pulmonary Associates, Corpus Christi, Texas, 78413, USA; 4 Weil Cornell University Houston Methodist Hospital, Houston Texas, 77030, USA; 5 The University of Texas Health Science Center at Houston, University of Texas, Medical Branch at Galveston, Chief of Critical Care Services, United Memorial Medical Center / United General Hospital, Houston, Texas, USA

**Keywords:** Vaccines, mRNA Vaccines, SARS-CoV-2, COVID-19, Cerebral venous embolism, Delta variant, Myocarditis

## Abstract

The emergence of SARS-CoV-2 in late December 2019 has taken the world by storm. In March 2020, the World Health Organization (WHO) named this virus COVID-19. To date, it has infected approximately 186 million people worldwide and is attributed as the cause of death of more than 5 million people (and this number is only increasing.) The global effort to develop vaccines and therapeutics occurred at the fastest pace yet, with several vaccines' approval under emergency authorization use. There are also several post-marketing side effects, including myocarditis, cerebral venous embolism, and Guillain Barre Syndrome. Global vaccine disparity complicates the control of pandemic challenges. Several highly infectious variants have emerged, and more variants are feared to emerge if global vaccination plans are not developed soon.

The COVID-19 pandemic, which continues to infect human lives across the globe, is far from over. The Severe Acute Respiratory Syndrome Coronavirus-2 (SARS-CoV-2) first emerged in Wuhan, Hubei province in China in December of 2019. Within a matter of months, by March 2020, it was already labeled a pandemic [1]. So far, it has infected more than 234 million people worldwide and has claimed the lives of more than 5 million people across the globe [2]. In the United States, the virus has infected more than 48 million people, followed by India with 34 million infected, and Brazil with more than 22 million people infected [2]. The world has moved unprecedentedly towards finding a cure for the disease in areas of both therapeutics and vaccines. With the success of previous vaccines, countries aggressively started moving towards the development of vaccines to offer the world hope and optimism. Usually, the vaccine process can take up to 8-10 years. Despite this norm, the pandemic made scientists and regulators work fast to develop vaccines in the shortest possible time. The virus challenged this success with the development of a multitude of variants. Overall, the vaccine development process goes through the pharmacological quality, non-clinical trials *in vitro* and vivo, clinical phase I, II, and III trials, evaluation and decision making, manufacturing and post-marketing safety survey, and phase IV trials. Details of Phases I, II, III, and IV are shown in Fig. (**1**). Fig. (**2**) illustrates the accelerated path of the vaccine development in COVID times compared to the normal development of the COVID vaccine in due course [3]. Several different technologies have been used to develop these vaccines. Some of them are new, while others have utilized older proven technologies [4]. The details of those vaccines are shown in Fig. (**3**) [4].

In the race of vaccine development to halt the rapid progression of the virus, which is causing an increase in morbidity and mortality, Phase II and III trials were run concurrently [5]. Vaccine production started even before trial results were completed to be ahead of the curve and get the vaccines to the global population as soon as possible. Every developed and developing country was pouring a significant amount of its budget towards securing vaccines. Vaccines manufacturers were taking pre-orders even before any trial results were out. Countries are in a race to get the best vaccines for their populations while forgetting this is a pandemic and not an endemic. In the USA, the messenger RNA-based technology vaccine from Pfizer and Moderna was selected as the silver bullet to end suffering, whereas, in the UK, the Oxford/AstraZeneca adenovirus-based vaccines were the frontrunners. Soon afterwards, the Johnson & Johnson single dose vaccine received emergency use authorization. The efficacy of the most common vaccines used in North America and Europe is shown in Fig. (**4**) [6, 7]. The Russian vaccine Sputnik and Chinese vaccines Cansino and Sinopharm were making headways in their respective countries and across other countries in Asia. Developed countries, including the USA and UK, were more focused on their vaccine procurement efforts. Under Operation Warp speed, the USA focused on getting 300 million doses of the vaccine for the prevention of COVID-19 infection by January 2021-a very optimistic goal to achieve what, on average, takes 73+ months. Operation Warp Speed spent 12.4 billion on vaccines by December 2020. Details of investment in different vaccine manufacturers are shown in Fig. (**5**) [8]. There were many critical and affirming statements regarding the amount of money spent on vaccine procurement. Still, when looking at the bigger picture concerning how many dollars were spent in other areas, this amount appeared to be minuscule, citing how the USA initially paid not enough healthcare dollars in vaccine procurement [8]. Many developing countries relied on the combined global effort through GAVI to guarantee at least 20% of needed vaccines for each country so frontline workers and high risk populations could be vaccinated [9]. The COVAX was launched in April 2020 to bring the private sector, nonprofit organizations, and government together to provide equitable solutions and access to COVID-19 vaccines, diagnostics, and therapeutics, offering hope to developing countries with access to vaccines, regardless of their financial capacities. Their goal is to coordinate with GAVI, WHO, Coalition for Epidemic Preparedness Innovations (CEPI), and vaccine alliance to coordinate with the manufacturers, negotiate prices, secure vaccines, and provide equal access to participating countries. Their goal is to have 2 billion vaccines by the end of 2021 and provide vaccines to high-risk populations and frontline workers [9]. The fundamental goal of GAVI and COVAX is to guarantee that the vaccine is accessible to the 92 low- and middle-income countries that cannot afford to vaccinate their people [9].

The COVAX prioritized investing in AstraZeneca, Johnson & Johnson, and the Novavax vaccine. They avoided mRNA-based vaccines due to cold chain technology issues, especially in developing countries and rural areas where vaccine delivery can be challenging. Vaccine producers like the Serum Institute of India and other significant global laboratories were contacted by vaccine developers to produce vaccines on a large scale.Several vaccines began to emerge, despite the fact that they had not yet been published in peer-reviewed journals [10].

While the USA and some developing countries started receiving vaccines (without knowing the long-term side effects and long-term efficacies) [11], the world patiently waited for their turn to be vaccinated. While vaccines from different companies were being delivered in small assignments, the world was simultaneously plagued with variants. Initially, the B.1.1.7 variant (Alpha Variant) in the UK and the South African and Brazilian variants emerged [12-14]. Vaccine developers were once again put on the spot, and they were compelled to shift their attention to conducting follow-up studies or enrolling patients in their studies in high-risk countries to demonstrate their vaccines' effectiveness against these variants.According to WHO, 102 vaccines are in clinical development and 185 vaccines are in pre-clinical development [15].

While vaccine development and distribution are in progress, different regions are differently impacted by the virus and its variants. While the USA and European nations are returning to their normal lives, the emergence of the Delta variant is plaguing India severely, completely collapsing their healthcare system. With the Delta variant relentlessly infecting the global population, it now accounts for more than 50% of new COVID-19 cases [12, 13]. With the Delta variant’s emergence, global numbers for COVID-19 positive cases have started increasing again, especially among non-vaccinated patients. The R0 factor for the Delta variant is in the range of 4-9. Compared to the wild variant R0 of 2.7, the mortality rate among patients infected with the Delta variant has also been reported significantly higher than the initial virus [16, 17]. The recent report also suggested the efficacy of the Sinopharm vaccine against the Delta variant [18].

The Lamda variant emerged in Peru in December 2020 and accounts for 80% of cases within the country. It has now spread to 26 countries. In June 2021, WHO recognized this variant but has yet to classify it as a variant of concern [19]. The Delta variant is now recognized as one of the most highly transmissible variants. Details of different variants are seen in Fig. (**6**).

The real-world data of the efficacy of the mRNA vaccine was in the range of above 90% for mild-severe infection, hospitalization, and ICU admissions. In comparison, with the emergence of the Delta variation, the Pfizer vaccine's effectiveness has dropped to 63% but remains over 90% for severe illness, hospitalization, and ICU admissions.The efficacy of the other non-mRNA vaccines has been reported to be much lower, making a call for the booster dose with mRNA vaccines or the second/third dose of the same vaccine if the mRNA vaccine is unavailable.

Treating COVID-19 as an isolated virus that can be prevented without global collaboration has resulted in the emergence and surge of more variants, especially in developed countries. The multiple global surges in COVID-19 cases are shown in Fig. (**7**) [2].

As we gather more data on vaccine safety and efficacy, we have learned about myocarditis in the mRNA-based vaccine [20], cerebral venous embolism in the adenovirus-based vaccine (AstraZeneca and J&J Vaccine) [21], and Guillan Barrie Syndrome (J&J Vaccine) [22]. The data on other vaccine efficacies, especially with Chinese and Russian-based vaccines, is addressed with less transparency.

The scientific success of developing an effective vaccine against COVID-19 has been overshadowed by poor vaccination rates, especially in developed countries. Mixed messages, politicization of vaccines, and misinformation repeated in social media lead to public distrust regarding vaccines. This has led to an increase in anti-vaxxers. Even though there is a plethora of data about the safety of the vaccine, politicization and twisted messages have gone viral, creating more challenges to achieve herd immunity.

This pandemic has not only caused high global infection rates and mortality but has caused a tremendous global economic toll. The disparity in the availability and distribution of vaccines has left developing nations at the mercy of the SARS-CoV-2 virus. While developed countries believed that vaccinating a significant proportion of their population would quickly lead to the opening of businesses, the economy, and borders, this approach backfired as variants began to emerge in other countries with low vaccination rates.These variants inevitably made it to developed countries. According to the Rockefeller Foundation, “The inequity and the lack of strong global vaccination campaigns extend beyond a health crisis. Our interconnected global economy stands to lose as much as US$9.2 trillion if governments fail to ensure developing economy access to COVID-19 vaccines” [23].

## CONCLUSION

To conclude, the death and infection toll should be kept minimal and the global economy should be safeguarded if the world wants to overcome this pandemic and prepare for future pandemics. To prevent contagious and non-infectious natural disasters, world leaders must sit down, coordinate, and work toward a global plan. With this pandemic, no one is safe until everyone is safe.

## Figures and Tables

**Fig. (1):**
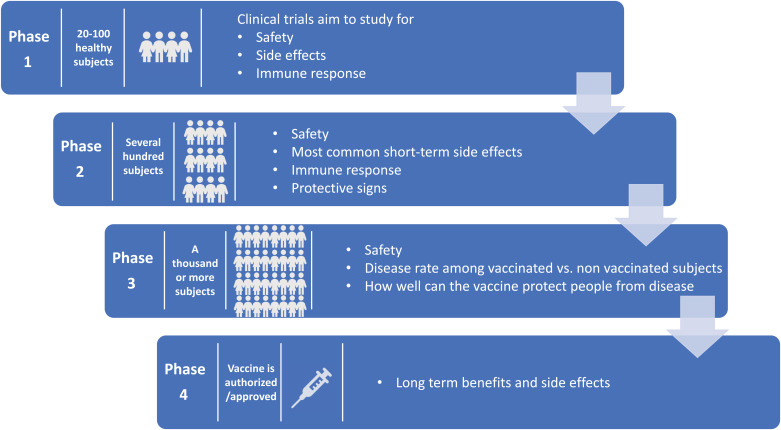
Showing different phases of clinical trials.

**Fig. (2):**
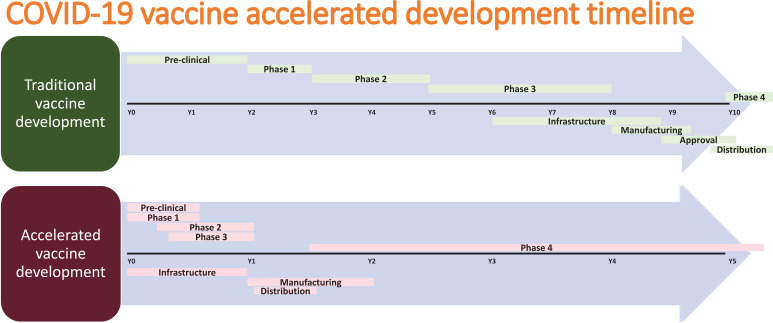
Accelerated pathway of vaccine development in covid times.

**Fig. (3):**
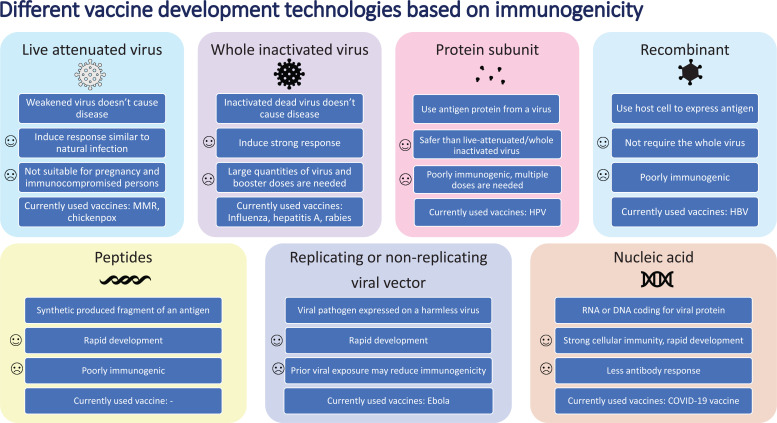
Vaccine development technology based on immunogenicity.

**Fig. (4):**
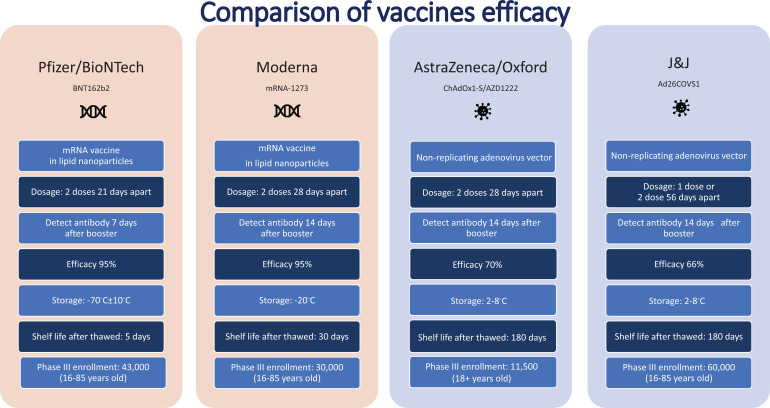
Efficacy of common vaccines used in USA and Europe.

**Fig. (5):**
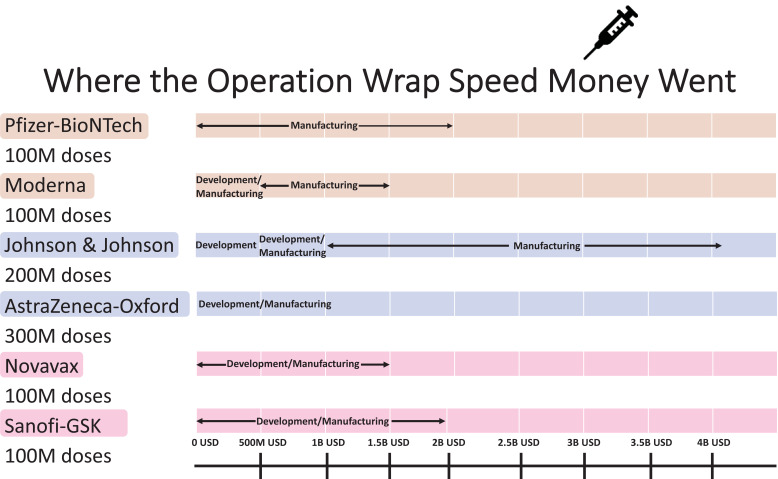
Where the operation wrap speed money went.

**Fig. (6):**
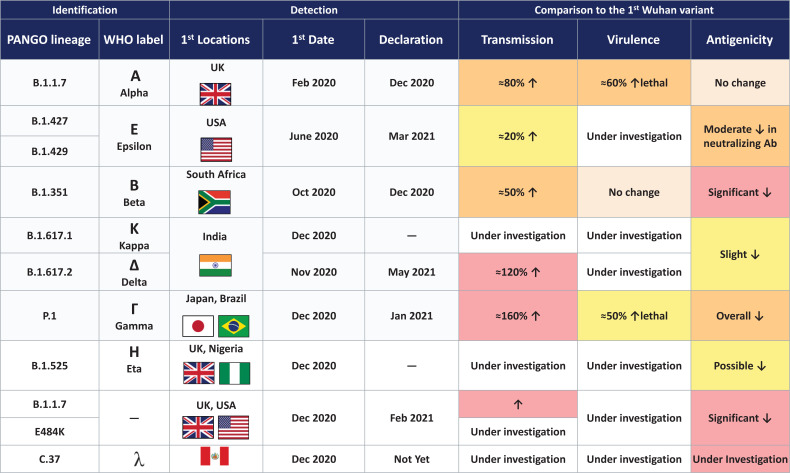
Multiple variants of concern.

**Fig. (7):**
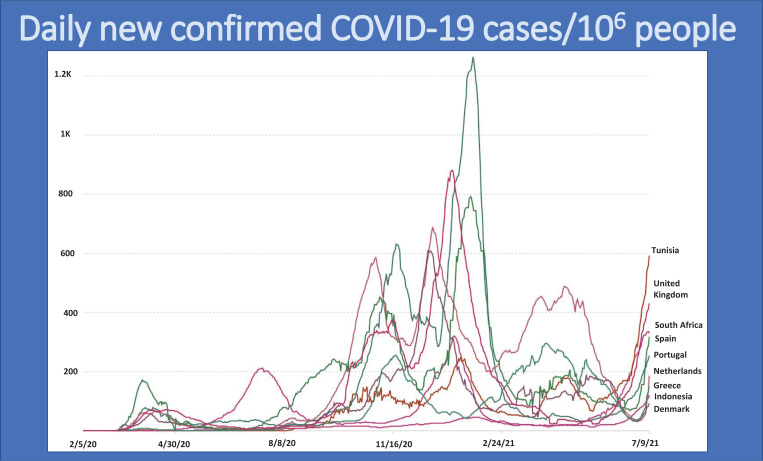
Global surges in COVID-19 cases. Source: https://ourworldindata.org/grapher/daily-covid-cases-deaths?country=~OWID_WRL.

## References

[1] Ochani R., Asad A., Yasmin F., Shaikh S., Khalid H., Batra S., Sohail M.R., Mahmood S.F., Ochani R., Hussham Arshad M., Kumar A., Surani S. (2021). COVID-19 pandemic: from origins to outcomes. A comprehensive review of viral pathogenesis, clinical manifestations, diagnostic evaluation, and management.. Infez. Med..

[2] COVID Live Update (2021). John Hopkins.

[3] Update 45, Vaccine development: World Health Organization (2021). https://www.who.int/docs/default-source/coronaviruse/risk-comms-updates/update45-vaccines-developement.pdf?sfvrsn=13098bfc_5.

[4] Dai L., Gao G.F. (2021). Viral targets for vaccines against COVID-19.. Nat. Rev. Immunol..

[5] Oxford COVID-19 vaccine to begin phase II/III human trials UK: OXford Press (2020). https://www.ox.ac.uk/news/2020-05-22-oxford-COVID-19-vaccine-begin-phase-iiiii-human-trials.

[6] Comparing the COVID-19 vaccines developed by Pfizer, Moderna, and Johnson & Johnson: Health (2021). https://www.statnews.com/2021/02/02/comparing-the-COVID-19-vaccines-developed-by-pfizer-moderna-and-johnson-johnson/.

[7] Voysey M., Clemens S.A.C., Madhi S.A., Weckx L.Y., Folegatti P.M., Aley P.K., Angus B., Baillie V.L., Barnabas S.L., Bhorat Q.E., Bibi S., Briner C., Cicconi P., Collins A.M., Colin-Jones R., Cutland C.L., Darton T.C., Dheda K., Duncan C.J.A., Emary K.R.W., Ewer K.J., Fairlie L., Faust S.N., Feng S., Ferreira D.M., Finn A., Goodman A.L., Green C.M., Green C.A., Heath P.T., Hill C., Hill H., Hirsch I., Hodgson S.H.C., Izu A., Jackson S., Jenkin D., Joe C.C.D., Kerridge S., Koen A., Kwatra G., Lazarus R., Lawrie A.M., Lelliott A., Libri V., Lillie P.J., Mallory R., Mendes A.V.A., Milan E.P., Minassian A.M., McGregor A., Morrison H., Mujadidi Y.F., Nana A., O’Reilly P.J., Padayachee S.D., Pittella A., Plested E., Pollock K.M., Ramasamy M.N., Rhead S., Schwarzbold A.V., Singh N., Smith A., Song R., Snape M.D., Sprinz E., Sutherland R.K., Tarrant R., Thomson E.C., Török M.E., Toshner M., Turner D.P.J., Vekemans J., Villafana T.L., Watson M.E.E., Williams C.J., Douglas A.D., Hill A.V.S., Lambe T., Gilbert S.C., Pollard A.J., Aban M., Abayomi F., Abeyskera K., Aboagye J., Adam M., Adams K., Adamson J., Adelaja Y.A., Adewetan G., Adlou S., Ahmed K., Akhalwaya Y., Akhalwaya S., Alcock A., Ali A., Allen E.R., Allen L., Almeida T.C.D.S.C., Alves M.P.S., Amorim F., Andritsou F., Anslow R., Appleby M., Arbe-Barnes E.H., Ariaans M.P., Arns B., Arruda L., Azi P., Azi L., Babbage G., Bailey C., Baker K.F., Baker M., Baker N., Baker P., Baldwin L., Baleanu I., Bandeira D., Bara A., Barbosa M.A.S., Barker D., Barlow G.D., Barnes E., Barr A.S., Barrett J.R., Barrett J., Bates L., Batten A., Beadon K., Beales E., Beckley R., Belij-Rammerstorfer S., Bell J., Bellamy D., Bellei N., Belton S., Berg A., Bermejo L., Berrie E., Berry L., Berzenyi D., Beveridge A., Bewley K.R., Bexhell H., Bhikha S., Bhorat A.E., Bhorat Z.E., Bijker E., Birch G., Birch S., Bird A., Bird O., Bisnauthsing K., Bittaye M., Blackstone K., Blackwell L., Bletchly H., Blundell C.L., Blundell S.R., Bodalia P., Boettger B.C., Bolam E., Boland E., Bormans D., Borthwick N., Bowring F., Boyd A., Bradley P., Brenner T., Brown P., Brown C., Brown-O’Sullivan C., Bruce S., Brunt E., Buchan R., Budd W., Bulbulia Y.A., Bull M., Burbage J., Burhan H., Burn A., Buttigieg K.R., Byard N., Cabera Puig I., Calderon G., Calvert A., Camara S., Cao M., Cappuccini F., Cardoso J.R., Carr M., Carroll M.W., Carson-Stevens A., Carvalho Y.M., Carvalho J.A.M., Casey H.R., Cashen P., Castro T., Castro L.C., Cathie K., Cavey A., Cerbino-Neto J., Chadwick J., Chapman D., Charlton S., Chelysheva I., Chester O., Chita S., Cho J-S., Cifuentes L., Clark E., Clark M., Clarke A., Clutterbuck E.A., Collins S.L.K., Conlon C.P., Connarty S., Coombes N., Cooper C., Cooper R., Cornelissen L., Corrah T., Cosgrove C., Cox T., Crocker W.E.M., Crosbie S., Cullen L., Cullen D., Cunha D.R.M.F., Cunningham C., Cuthbertson F.C., Da Guarda S.N.F., da Silva L.P., Damratoski B.E., Danos Z., Dantas M.T.D.C., Darroch P., Datoo M.S., Datta C., Davids M., Davies S.L., Davies H., Davis E., Davis J., Davis J., De Nobrega M.M.D., De Oliveira Kalid L.M., Dearlove D., Demissie T., Desai A., Di Marco S., Di Maso C., Dinelli M.I.S., Dinesh T., Docksey C., Dold C., Dong T., Donnellan F.R., Dos Santos T., dos Santos T.G., Dos Santos E.P., Douglas N., Downing C., Drake J., Drake-Brockman R., Driver K., Drury R., Dunachie S.J., Durham B.S., Dutra L., Easom N.J.W., van Eck S., Edwards M., Edwards N.J., El Muhanna O.M., Elias S.C., Elmore M., English M., Esmail A., Essack Y.M., Farmer E., Farooq M., Farrar M., Farrugia L., Faulkner B., Fedosyuk S., Felle S., Feng S., Ferreira Da Silva C., Field S., Fisher R., Flaxman A., Fletcher J., Fofie H., Fok H., Ford K.J., Fowler J., Fraiman P.H.A., Francis E., Franco M.M., Frater J., Freire M.S.M., Fry S.H., Fudge S., Furze J., Fuskova M., Galian-Rubio P., Galiza E., Garlant H., Gavrila M., Geddes A., Gibbons K.A., Gilbride C., Gill H., Glynn S., Godwin K., Gokani K., Goldoni U.C., Goncalves M., Gonzalez I.G.S., Goodwin J., Goondiwala A., Gordon-Quayle K., Gorini G., Grab J., Gracie L., Greenland M., Greenwood N., Greffrath J., Groenewald M.M., Grossi L., Gupta G., Hackett M., Hallis B., Hamaluba M., Hamilton E., Hamlyn J., Hammersley D., Hanrath A.T., Hanumunthadu B., Harris S.A., Harris C., Harris T., Harrison T.D., Harrison D., Hart T.C., Hartnell B., Hassan S., Haughney J., Hawkins S., Hay J., Head I., Henry J., Hermosin Herrera M., Hettle D.B., Hill J., Hodges G., Horne E., Hou M.M., Houlihan C., Howe E., Howell N., Humphreys J., Humphries H.E., Hurley K., Huson C., Hyder-Wright A., Hyams C., Ikram S., Ishwarbhai A., Ivan M., Iveson P., Iyer V., Jackson F., De Jager J., Jaumdally S., Jeffers H., Jesudason N., Jones B., Jones K., Jones E., Jones C., Jorge M.R., Jose A., Joshi A., Júnior E.A.M.S., Kadziola J., Kailath R., Kana F., Karampatsas K., Kasanyinga M., Keen J., Kelly E.J., Kelly D.M., Kelly D., Kelly S., Kerr D., Kfouri R.Á., Khan L., Khozoee B., Kidd S., Killen A., Kinch J., Kinch P., King L.D.W., King T.B., Kingham L., Klenerman P., Knapper F., Knight J.C., Knott D., Koleva S., Lang M., Lang G., Larkworthy C.W., Larwood J.P.J., Law R., Lazarus E.M., Leach A., Lees E.A., Lemm N-M., Lessa A., Leung S., Li Y., Lias A.M., Liatsikos K., Linder A., Lipworth S., Liu S., Liu X., Lloyd A., Lloyd S., Loew L., Lopez Ramon R., Lora L., Lowthorpe V., Luz K., MacDonald J.C., MacGregor G., Madhavan M., Mainwaring D.O., Makambwa E., Makinson R., Malahleha M., Malamatsho R., Mallett G., Mansatta K., Maoko T., Mapetla K., Marchevsky N.G., Marinou S., Marlow E., Marques G.N., Marriott P., Marshall R.P., Marshall J.L., Martins F.J., Masenya M., Masilela M., Masters S.K., Mathew M., Matlebjane H., Matshidiso K., Mazur O., Mazzella A., McCaughan H., McEwan J., McGlashan J., McInroy L., McIntyre Z., McLenaghan D., McRobert N., McSwiggan S., Megson C., Mehdipour S., Meijs W., Mendonça R.N.Á., Mentzer A.J., Mirtorabi N., Mitton C., Mnyakeni S., Moghaddas F., Molapo K., Moloi M., Moore M., Moraes-Pinto M.I., Moran M., Morey E., Morgans R., Morris S., Morris S., Morris H.C., Morselli F., Morshead G., Morter R., Mottal L., Moultrie A., Moya N., Mpelembue M., Msomi S., Mugodi Y., Mukhopadhyay E., Muller J., Munro A., Munro C., Murphy S., Mweu P., Myasaki C.H., Naik G., Naker K., Nastouli E., Nazir A., Ndlovu B., Neffa F., Njenga C., Noal H., Noé A., Novaes G., Nugent F.L., Nunes G., O’Brien K., O’Connor D., Odam M., Oelofse S., Oguti B., Olchawski V., Oldfield N.J., Oliveira M.G., Oliveira C., Oosthuizen A., O’Reilly P., Osborne P., Owen D.R.J., Owen L., Owens D., Owino N., Pacurar M., Paiva B.V.B., Palhares E.M.F., Palmer S., Parkinson S., Parracho H.M.R.T., Parsons K., Patel D., Patel B., Patel F., Patel K., Patrick-Smith M., Payne R.O., Peng Y., Penn E.J., Pennington A., Peralta Alvarez M.P., Perring J., Perry N., Perumal R., Petkar S., Philip T., Phillips D.J., Phillips J., Phohu M.K., Pickup L., Pieterse S., Piper J., Pipini D., Plank M., Du Plessis J., Pollard S., Pooley J., Pooran A., Poulton I., Powers C., Presa F.B., Price D.A., Price V., Primeira M., Proud P.C., Provstgaard-Morys S., Pueschel S., Pulido D., Quaid S., Rabara R., Radford A., Radia K., Rajapaska D., Rajeswaran T., Ramos A.S.F., Ramos Lopez F., Rampling T., Rand J., Ratcliffe H., Rawlinson T., Rea D., Rees B., Reiné J., Resuello-Dauti M., Reyes Pabon E., Ribiero C.M., Ricamara M., Richter A., Ritchie N., Ritchie A.J., Robbins A.J., Roberts H., Robinson R.E., Robinson H., Rocchetti T.T., Rocha B.P., Roche S., Rollier C., Rose L., Ross Russell A.L., Rossouw L., Royal S., Rudiansyah I., Ruiz S., Saich S., Sala C., Sale J., Salman A.M., Salvador N., Salvador S., Sampaio M., Samson A.D., Sanchez-Gonzalez A., Sanders H., Sanders K., Santos E., Santos Guerra M.F.S., Satti I., Saunders J.E., Saunders C., Sayed A., Schim van der Loeff I., Schmid A.B., Schofield E., Screaton G., Seddiqi S., Segireddy R.R., Senger R., Serrano S., Shah R., Shaik I., Sharpe H.E., Sharrocks K., Shaw R., Shea A., Shepherd A., Shepherd J.G., Shiham F., Sidhom E., Silk S.E., da Silva Moraes A.C., Silva-Junior G., Silva-Reyes L., Silveira A.D., Silveira M.B.V., Sinha J., Skelly D.T., Smith D.C., Smith N., Smith H.E., Smith D.J., Smith C.C., Soares A., Soares T., Solórzano C., Sorio G.L., Sorley K., Sosa-Rodriguez T., Souza C.M.C.D.L., Souza B.S.D.F., Souza A.R., Spencer A.J., Spina F., Spoors L., Stafford L., Stamford I., Starinskij I., Stein R., Steven J., Stockdale L., Stockwell L.V., Strickland L.H., Stuart A.C., Sturdy A., Sutton N., Szigeti A., Tahiri-Alaoui A., Tanner R., Taoushanis C., Tarr A.W., Taylor K., Taylor U., Taylor I.J., Taylor J., te Water Naude R., Themistocleous Y., Themistocleous A., Thomas M., Thomas K., Thomas T.M., Thombrayil A., Thompson F., Thompson A., Thompson K., Thompson A., Thomson J., Thornton-Jones V., Tighe P.J., Tinoco L.A., Tiongson G., Tladinyane B., Tomasicchio M., Tomic A., Tonks S., Towner J., Tran N., Tree J., Trillana G., Trinham C., Trivett R., Truby A., Tsheko B.L., Turabi A., Turner R., Turner C., Ulaszewska M., Underwood B.R., Varughese R., Verbart D., Verheul M., Vichos I., Vieira T., Waddington C.S., Walker L., Wallis E., Wand M., Warbick D., Wardell T., Warimwe G., Warren S.C., Watkins B., Watson E., Webb S., Webb-Bridges A., Webster A., Welch J., Wells J., West A., White C., White R., Williams P., Williams R.L., Winslow R., Woodyer M., Worth A.T., Wright D., Wroblewska M., Yao A., Zimmer R., Zizi D., Zuidewind P., Oxford COVID Vaccine Trial Group (2021). Safety and efficacy of the ChAdOx1 nCoV-19 vaccine (AZD1222) against SARS-CoV-2: an interim analysis of four randomised controlled trials in Brazil, South Africa, and the UK.. Lancet.

[8] (2020). Times. The trump administration's 'operation warp speed' has spent $12.4 billion on vaccines.. How Much Is That, Really?.

[9] alliance GTv (2021). COVAX Explained.

[10] Cui X., Wang P., Wei Z. (2021). Emergency use of COVID-19 vaccines recommended by the World Health Organization (WHO) as of June 2021.. Drug Discov. Ther..

[11] Katella K., Available from:
https://www.yalemedicine.org
/news/COVID-19-vaccine-comparison (2021). Comparing the COVID-19 vaccines: How are they different.. Yale Medicine.

[12] Lauring A.S., Hodcroft E.B. (2021). Genetic variants of SARS-CoV-2—what do they mean?. JAMA.

[13] Vasireddy D., Vanaparthy R., Mohan G., Malayala S.V., Atluri P. (2021). Review of COVID-19 variants and covid-19 vaccine efficacy: What the clinician should know?. J. Clin. Med. Res..

[14] Vasireddy D., Atluri P., Malayala S.V., Vanaparthy R., Mohan G. (2021). Review of COVID-19 vaccines approved in the united states of america for emergency Use.. J. Clin. Med. Res..

[15] COVID-19 vaccine tracker and landscape: WHO (2021). https://www.who.int/publications/m/item/draft-landscape-of-COVID-19-candidate-vaccines.

[16] Farinholt T., Doddapaneni H., Qin X., Menon V., Meng Q., Metcalf G. (2021). Transmission event of SARS-CoV-2 Delta variant reveals multiple vaccine breakthrough infections.. medRxiv.

[17] Delta Variant T.P. (2021). Everything you need to know.

[18] (2021). Sinopharm vaccine effective against coronavirus Delta variant, study finds
: N UAE.

[19] Wikipedia (2021). SARS-CoV-2 Lambda variant.

[20] Singh B., Kaur P., Cedeno L., Brahimi T., Patel P., Virk H., Shamoon F., Bikkina M. (2021). COVID-19 mRNA Vaccine and Myocarditis.. Eur. J. Case Rep. Intern. Med..

[21] Wang R.L., Chiang W.F., Shyu H.Y., Chen M.H., Lin C.I., Wu K.A., Yang C.C., Huang L.Y., Hsiao P.J. (2021). COVID-19 vaccine-associated acute cerebral venous thrombosis and pulmonary artery embolism.. QJM.

[22] Letter to Janseen Inc (2021). Health & Human Services.

[23] Foundation R. (2021). One for all: An updated action plan for global COVID-19 vaccination.

